# Plasma fibrinogen in the diagnosis of periprosthetic joint infection

**DOI:** 10.1038/s41598-020-80547-z

**Published:** 2021-01-12

**Authors:** Fei Yang, Chenyu Zhao, Rong Huang, Hui Ma, Xiaohe Wang, Guodong Wang, Xiaowei Zhao

**Affiliations:** 1 Department of Orthopedics, The People’s Hospital of Bozhou, Bozhou, 236800 Anhui China; 2 Department of Orthopedics, The Affiliated Hospital of Jining Medical University, 89 Guhuai Road, Jining, 272067 Shandong China; 3 West China School of Basic Medical Science & Forensic Medicine, Sichuan University, Chendu, 610041 China

**Keywords:** Diagnostic markers, Infectious diseases, Pain

## Abstract

Periprosthetic joint infections (PJIs) have become the most catastrophic complication for patients after arthroplasty. Although previous studies have found that many biomarkers have good performance for diagnosing PJI, early diagnosis remains challenging and a gold standard is lacking. This study aimed to investigate the diagnostic accuracy of plasma fibrinogen (FIB) in detecting PJI compared to other traditional biomarks (CRP, WBC and ESR). A total of 156 patients (including 57 PJI and 99 non-PJI patients) who underwent revision arthroplasty were retrospectively reviewed from 01/2014 to 01/2020. The diagnostic criteria of PJI were mainly based on the definition from the evidence-based definition for periprosthetic joint infection in 2018. The optimal plasma FIB predictive cutoff was 4.20 g/L, the sensitivity of the plasma fibrinogen was 0.860, the specificity was 0.900, the positive predictive value (PPV) was 0.831, and the negative predictive value (NPV) was 0.908. The area under the curve (AUC) value of plasma fibrinogen was 0.916 (95% CI 0.869–0.964), and the CRP, ESR and WBC levels had AUCs of 0.901, 0.822 and 0.647, respectively. Plasma FIB demonstrated better diagnostic strength compared with that of other serum biomarkers before revision arthroplasty. It represents a new horizon for the diagnosis of PJI due to the diagnosis values and cost-effective features.

Periprosthetic joint infections (PJIs) have become the most catastrophic complication after total hip and knee arthroplasty and are a tremendous burden for patients and institutions worldwide. Approximately 1% of patients who underwent hip arthroplasties and 1–2% of patients who underwent knee arthroplasties^1–3^ experienced PJI. Previous research has established that PJI was the most common revision reason for knee and the fourth most common revision reason for hip^4,5^. Early and correct diagnosis is particularly critical for treatment^6^. Although the MSIS has recommended that the clinical features, serum and synovial fluid biomarkers, microbiology, and histopathology, in previous studies have provided important information on new biomarkers (e.g., alpha-defensin, procalcitonin, IL-6, nuclear imaging techniques), the early diagnosis remains challenging and lacks a gold standard^7–10^.

Plasma FIB is one of the coagulation-related indicators and is traditionally used in venous thrombus embolism (VTE), it is particularly critical for regulating inflammation of infections^11,12^. Previous studies revealed that plasma fibrinogen was significantly associated with the diagnosis of PJI. Xu et al.^13^ first demonstrated that plasma fibrinogen levels were higher in PJI than in aseptic failure. However, only a few studies have further confirmed the value of plasma fibrinogen, and the diagnostic accuracy remains unknown.

The purpose of this study was to evaluate the optimal cutoff and diagnosis values of plasma FIB in PJI and to compare them with the CRP, ESR and serum WBC count through a retrospective study.

## Methods

In the present study, we conducted a single-center, retrospective cohort study to investigate the diagnostic values of plasma FIB in PJI. This study was approved by Ethics Committee of the Affiliated Hospital of Jining Medical University. All methods were carried out in accordance with relevant guidelines and regulation and all patients signed informed consent before data collection. The data of patients in the database were anonymous for the purpose of protecting participants’ privacy, and the entire process of data collection was nonselective and consecutive. The data were obtained from the hospital electronic medical record system. The study initially collected a total of 189 patients who underwent revision total knee and hip arthroplasty from 01/2014 to 01/2020. The definition of PJI is completely in accordance with the evidence-based definition for periprosthetic joint infection in 2018 (Table 1).

**Table 1:** The evidence-based definition for periprosthetic joint infection (PJI).

	Diagnosis criteria	Score	Decision
Major criteria	1. Two positive cultures with the same organisms	–	Infected
	2. A sinus tract communicating with the joint	–	
Minor criteria	1. Elevated serum CRP or D-Dimer 2. Elevated serum ESR 3. Elevated synovial WBC or LE 4. Positive alpha-defensin 5. Elevated synovial PMN (%) 6. Elevated synovial CRP	2	≥ 6 infected 2–5 possibly infected 0–1 not infected
		1	
		3	
		3	
		2	
		1	
Inconclusive pro-op score or dry tap	1. Preoperative score 2. Positive histology 3. Positive purulence 4. Sing positive culture	–	≥ 6 infected 4–5 Inconclusive ≤ 3 not infected
		3	
		3	
		2	

**Figure 1:**
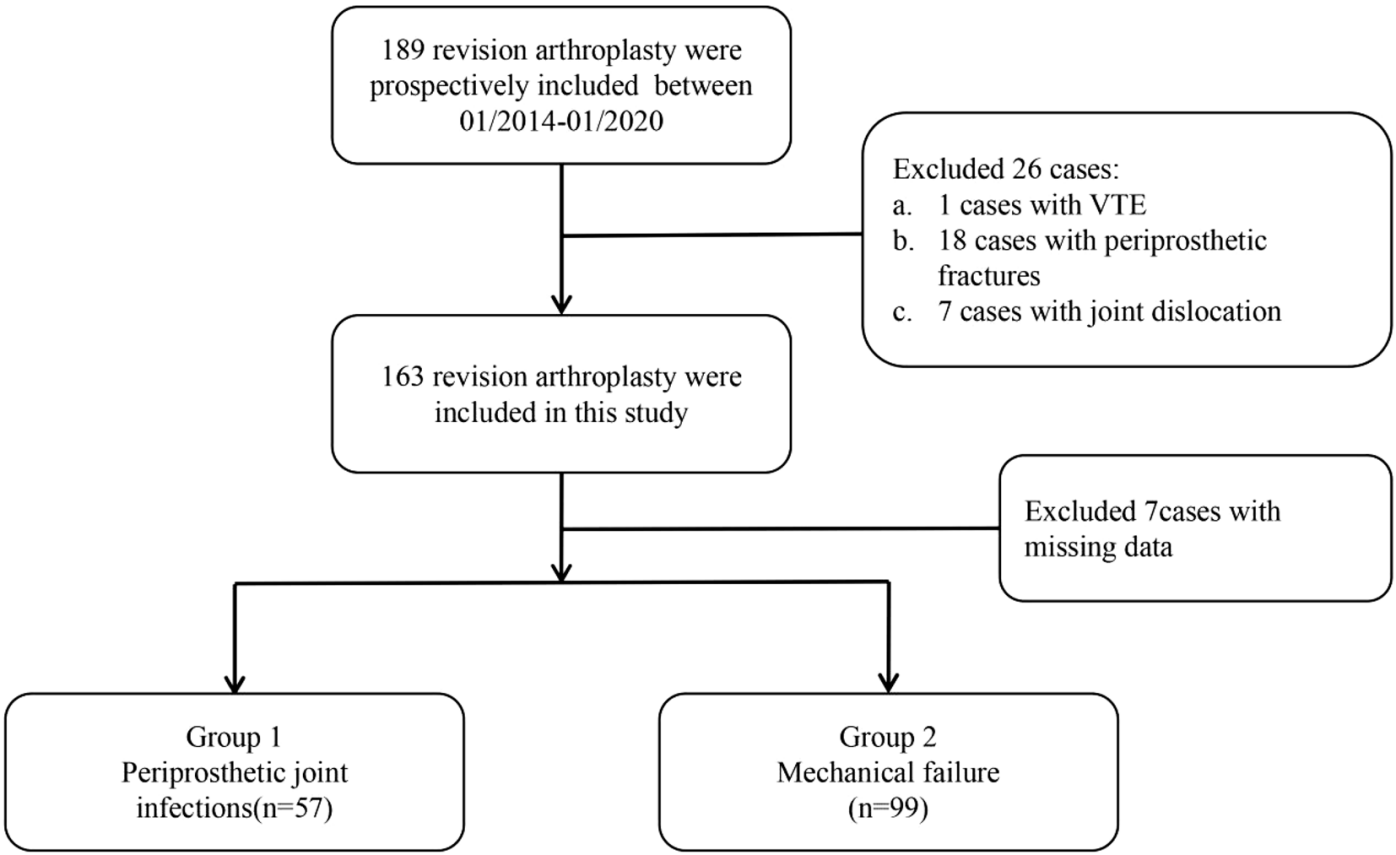
The inclusion and exclusion criteria of patients in the study design.

A total of 156 patients with revision total knee and hip arthroplasty due to infection and aseptic mechanical failure were included in the study. 1 patients with VTE was excluded, as well as 16 patients with periprosthetic fractures and 5 patients with joint dislocation. Additionally, patients with systemic lupus erythematosus, hepatitis B and C, gout, malignancy, systemic infection, and heavy smoking were also excluded (Fig. 1). The 156 cases were divided into 57 PJI cases and 99 cases non-PJI in the study using the modified criteria in 2018.

To detect the biomarkers, blood samples were collected from fasting patients on the morning after admission. All patients were routinely tested and analyzed for plasma fibrinogen, ESR, CRP, and serum WBC count. Synovial fluid samples were aspirated in the joint before surgery and aspirated during the revision procedure and were cultured. Periprosthetic tissues were sent for histologic analysis. The patient demographics, sex, age, infection position, body mass index (BMI), ASA score and the time to revision were collected from medical records.

Continuous variables were expressed as the mean ± standard deviation. All data analyses were performed with GraphPad Prism 8 (https://www.graphpad.com/). A T-test was carried out to compare significant differences. P-values < 0.05 were used to indicate statistically significant differences. Receiver operating characteristic (ROC) curves and the AUC were used to measure diagnostic values. The optimal predictive cutoffs were determined using the Youden index.

### Data availability

The datasets generated during and/or analyzed during the current study are available from the corresponding author on reasonable request.

## Results

**Figure 2:**
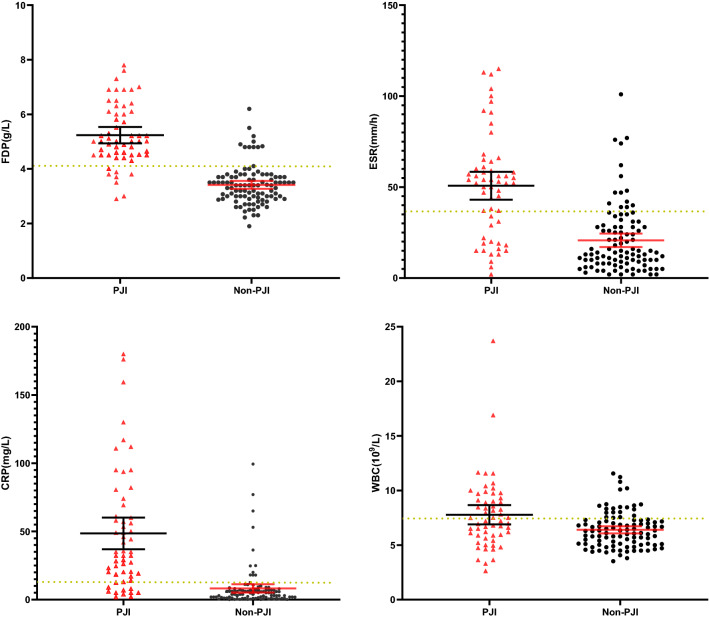
Analyses the distributions of plasma fibrinogen and ESR, CRP and serum WBC count. The solid line represents the average and the 95% CI and the dashed line indicates the optimal cutoff valves in the study, dotted lines represents the optimal cutoff values.

The basic characteristics of the patients are shown in Table 2. The results of these tested biomarkers are shown in Table 3. Compared with those in the non-PJI group, the plasma FIB, ESR, CRP and serum WBC counts of all the patients significantly increased in the PJI group (all P < 0.01). In particular, the plasma fibrinogen was significantly higher in the PJI group than that in the non-PJI group: 5.23 ± 1.09 vs 3.48 ± 0.82 g/L, and the distributions of these biomarkers are shown in Fig. 2.

**Table 2:** Basic characteristics of patients in the study.

Patients	PJI	Non-PJI	*P* value
Number	57 (36.5%)	99 (63.5%)	
**Gender**			0.964
Male	28 (49.1%)	49 (49.5%)	
Female	29 (50.9%)	50 (50.5%)	
Age (year)	65.4 ± 10.9 (27–88)	63.1 ± 10.0 (26–86)	0.163
BMI (kg/m^2^)	24.0 ± 4.29 (14.5–35.2)	25.6 ± 4.05 (16.6–38.7)	0.022
**Affected joint**			< 0.001
Knee	33 (57.9%)	17 (17.2%)	
Hip	24 (42.1%)	82 (82.8%)	
**Position**			0.032
Left	25 (43.9%)	61 (61.7%)	
Right	32 (56.1%)	38 (38.3%)	
**ASA classification**			0.777
1	3 (5.3%)	5 (5.1%)	
2	29 (50.1%)	47 (47.5%)	
3	21(36.8%)	43 (43.4%)	
4	4 (7.8%)	4 (4.0%)	
Time to revision (year)	3.3 ± 4.3 (0.05–17)	9.4 ± 6.2 (0.17–30)	< 0.001

**Table 3:** The traditional biomarkers and plasma fibrinogen between PJI group and non-PJI group.

Biomarks	PJI group	Non-PJI group	*P* value
Fibrinogen (g/L)	5.23 ± 1.12	3.41 ± 0.72	< 0.001
ESR (mm/h)	50.72 ± 28.60	20.74 ± 18.36	< 0.001
CRP (mg/L)	49.24 ± 45.77	6.67 ± 11.46	< 0.001
Serum WBC (10^9^/L)	7.78 ± 3.28	6.40 ± 1.62	< 0.001

**Figure 3:**
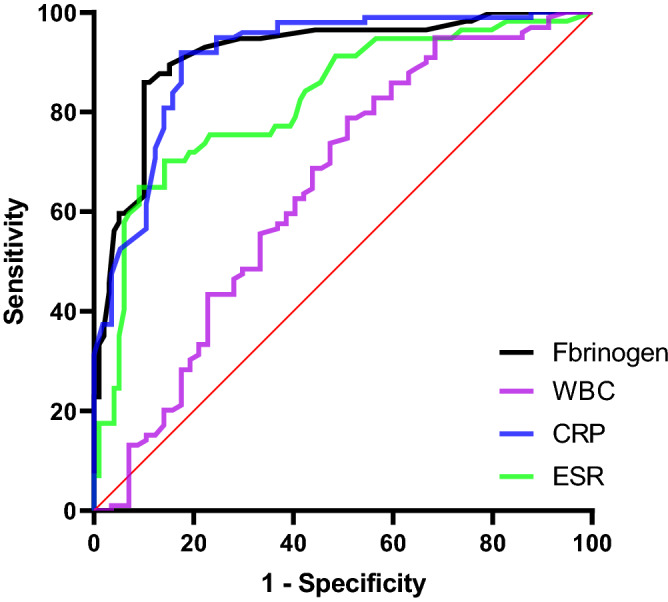
The ROC curves of plasma fibrinogen, ESR, CRP and serum WBC count for the diagnosis of PJI.

The AUCs were analyzed for the ROC curves (Fig. 3), and the results for plasma FIB, ESR, CRP and serum WBC count were 0.916 (95% CI 0.869–0.964), 0.822 (95% CI 0.752–0.893), 0.901 (95% CI 0.861–0.960) and 0.647 (95% CI 0.553–0.743), respectively (Table 4). The optimal cutoff was determined according to the Youden index using ROC curves. The calculated cutoff of plasma FIB was 4.20 g/L, and its sensitivity, specificity, PPV and NPV values were 0.860, 0.900, 0.831 and 0.908, respectively. With the calculated cutoff of CRP at 12.51 mg/L, the sensitivity, specificity, PPV, and NPV were 0.912, 0.827, 0.870, and 0.902, respectively. For ESR, the calculated cutoff was 36.50 mm/h, and the sensitivity, specificity, PPV, and NPV were 0.702, 0.859, 0.741, and 0.833, respectively. For the serum WBC count, the optimal cutoff was 7.42 × 10^9^/L, and the sensitivity, specificity, PPV, and NPV were 0.788, 0.492, 0.571, and 0.729, respectively (Table 4).

**Table 4:** The diagnosis values and threshold of plasma fibrinogen and ESR, CRP and serum WBC count for 156 cases.

Biomarks	AUC	95%CI	Youden Index	Threshold	Sensitivity	Specificity	PPV	NPV
Fibrinogen	0.916	0.869–0.964	0.759	4.20 (g/L)	0.860	0.900	0.831	0.908
ESR	0.822	0.752–0.893	0.561	36.50 (mm/h)	0.702	0.859	0.741	0.833
CRP	0.901	0.861–0.960	0.744	12.51 (mg/L)	0.912	0.827	0.870	0.902
WBC	0.647	0.553–0.743	0.280	7.42 (10^9^/L)	0.788	0.492	0.571	0.729

The culture results of the 57 PJI group are shown in Table 5. The main pathogens of PJI was *Staphylococcus aureus* in 11 patients (35.5%), followed by *Staphylococcus epidermidis* (29.0%) and *Candida parasitosis* (6.5%). In addition, 26 PJI patients had negative culture.

**Table 5:** Culture results of patients with PJI.

Culture results	Cases
**Positive**	31
*Staphylococcus aureus*	11
*Staphylococcus epidermidis*	9
*Candida parasilosis*	2
*Enterobacter cloacae*	1
*Propionibacterium acnes*	1
*Enterococcus faecalls*	1
*Stretococcus sanguinis*	1
Methicillin-resistant *Staphylococcus aureus* (MRSA)	1
*Mycobacterium tuberculosis*	1
*Finegoldia magna*	1
*Pseudomonas aeruginosa*	1
*Pseudomonas aeruginosa*	1
Negative	26

## Discussion

Although diagnosis methods of PJI have achieved rapid development in recent years and more and more new biomarkers and technologies have been developed, such as alpha-defensin, synovial fluid viscosity and D-lactate, 18F FDG-PET/CT and next-generation sequencing, the diagnosis of PJI remains extensively debated^14–18^. The guidelines are also constantly improving, and the Musculoskeletal Infection Society created the definition for PJI in 2011^19^. The ICM further modified the definition in 2013 and added the leukocyte esterase test in this modified guideline and standardized the acceptable threshold. In 2018, the definition for PJI was modified again; it added the D-dimer, synovial CRP and alpha-defensin in this modified guideline and assigned a score^20^. These modified criteria showed a higher sensitivity and specificity. Therefore, it is crucial for the diagnosis of PJI that more and more biomarkers with good diagnostic value be found.

The traditional biomarkers have been confirmed in previous studies to be reliable biomarkers and to have good sensitivity and specificity. ESR and CRP are the most commonly used classical markers in the diagnosis of PJI, with the best threshold of 30 mm/h and 10 mg/dL, and perform well for diagnosing PJI. The sensitivity of ESR ranged from 42 to 94%, and specificity ranged from 33 to 87%. On the other hand, the specificity of CRP ranged from 74 to 94%, and the specificity ranged from 20 to 100%^21^. Cipriano et al.^22^ showed that ESR and CRP have similar diagnostic values in both inflammatory and noninflammatory groups. Jane et al.^23^ found that ESR and CRP perform well in the nonobese group compared with the obese group with the same cutoff. Serum WBCs is commonly a marker of suspected cases in routine workups, doubts about the utility were raised regarding the diagnosis of PJI compared with synovial WBC. Toossi et al.^24^ found that the serum WBC count was significantly different between the PJI group and the non-PJI group (9236 cells/μL vs 7331 cells/μL), but its AUC, sensitivity and specificity values were 0.637, 0.55 and 0.66, respectively. The value of ESR, CRP and serum WBCs were limited. Compared with other biomarkers, the AUC of plasma FIB was increased in this study, revealing that it maybe performed well for the diagnosis of PJI. The AUCs of ESR and CRP levels were between 0.8 and 0.91, which indicates that the inflammatory status had good diagnostic value. In contrast, the AUC of serum WBC levels was 0.647 and indicates that serum WBCs had poor diagnostic value.

Recent evidence suggests that coagulation-related indicators may be promising markers for the diagnosis of PJI. Compared with other tests, the coagulation-related indicators are a simple, relatively inexpensive and noninvasive diagnostic method. In addition, the coagulation function test is a routine test after admission. The purpose of the coagulation system is hemostasis, and it is closely related to infection. The system can prevent the spread of the virus through the coagulation cascade^25^. Synovitis can generate lots of fibrin, and the degradation of fibrin will lead to elevated indicators; the indicators subsequently can have inflammatory effects^26^. It is possible that bacterial infections result in the activation of neutrophils and promote coagulation through tissue factor pathway inhibitor^27^. Tejbir et al.^28^ demonstrated that serum D-dimer has higher sensitivity and specificity and outperforms other conventional tests, such as ESR and CRP. Lee et al.^29^ found that D-dimer may serve as a new potential method for the early diagnosis of PJI due to its rapid changes and short half-life.

Plasma fibrinogen is a large (340 kDa) hexametric homodimer and is synthesized primarily in the liver; it can rapidly increase following injury and is essential for the infection process^30^. In addition, plasma fibrinogen is a better biomarker for COPD disease progression and wound healing and regulates nervous system functions^31,32^. Klim et al.^33^ found that plasma fibrinogen is a significant biomarker for detecting PJI due to a sensitivity of 0.90 and specificity of 0.66 through a prospective study. Li et al.^34^ have provided important information on serum fibrinogen, with 439 cases in which fibrinogen had the highest AUC of 0.852 and sensitivity of 0.763, specificity of 0.862, PPV of 0.537, and NPV of 0.946 and exhibited promising performance. In our study, we evaluated the baselines of 156 patients and the levels of plasma FIB in the PJI and non-PJI groups. Then, we compared the diagnostic accuracy of plasma fibrinogen with ESR, CRP and serum WBCs for PJI diagnosis. The plasma FIB exhibited promising performance, with an AUC of 0.916, a sensitivity of 0.860, and a specificity of 0.900. Our results are consistent with previous evidence that plasma FIB is an important biomarker for the diagnosis of PJI. The role of plasma FIB in judging whether the infection is cleared will be the content of our next study.

The majority of culture results of periprosthetic tissue were positive, the results of pathogens are consistent with previous evidence^35^. However, there are 26 patients had negative culture due to the culture time was short (only 7–10 days) and the majority of patients were referred to our center elsewhere and we don’t confirm the patients with previous antibiotic treatment.

There were some potential limitations in this study. First, the majority of patients were referred to our center from elsewhere, and we did not confirm the previous antibiotic treatment received by patients. Second, the cutoff and values of biomarkers were different between acute and chronic or hip and knee infections, and we could not conduct subgroup analysis due to the limited number of cases. Third, this study set strict inclusion and exclusion criteria, and the diagnostic value cannot be fully analyzed, which may overestimate the diagnostic accuracy of plasma fibrinogen.

## Conclusion

Considering the results and analyses mentioned above, this study further investigates the diagnostic value of plasma FIB in the diagnosis of PJI. The plasma FIB showed good performance and had the highest AUC and specificity compared with the traditional biomarkers. The roles of coagulation-related factors in the diagnosis of PJI should be more worthy of further studies and need more multi-institutional, large-scale, prospective, well-performed studies to improve the findings.
